# Case of Subclavian Aneurism, Which Terminated Favourably under Large and Repeated Bleedings—The Long-Continued Exhibition of Digitalis—Application of Pressure, &c. &c.

**Published:** 1815-05

**Authors:** John C. Yeatman

**Affiliations:** Member of the Royal College of Surgeons, &c. Frome


*For the Medical and Physical Journal.*

### *Case of Subclavian Aneurism, which terminated favourably under large and repeated Bleedings—The long-continued Exhibition of Digitalis—Application of Pressure, &c. &c.* By Mr. JOHN C. YEATMAN, of Frome.

DANIEL OWEN, living near Lawford's-gate, Bristol, æt. 37; robust, healthy from childhood; of a calm and firm mind; applied to me with an aneurism of the left subclavian artery.

This man has been employed in rope-making for five and twenty years. He perceived, about three months ago, a tumour of the size of a large nutmeg above the left clavicle, but did not think it of consequence to him. Was at this time employed in manufacturing and stretching some of the larger kind of rope. Had a hard cough and also a dull pain in the arm, which he attributed to rheumatism.

A month elapsed, when he discovered a pulsatory motion in the tumour; says it was evidently enlarged; felt at this period a tingling sensation in the fingers, with a dull pain

 and and numbness in the middle and outer part of the fore-arm, an inch below the origin of the brachialis internus muscle, which was occasionally suspended by friction.

February 20th, 1813.—The tumour, which appears to be of the size and shape of a pullet's egg, lies at the left side and root of the neck, just above the superior edge of the clavicle, and within an inch and a half of the sternal extremity of that bone, with its apex touching the edge of the outer head of the sterno cleido mastoideus; its base low down between the anterior and middle scaleni muscles, and its superior and external surface directly beneath the fibres of the platysma myoides. The tip of the little finger may be insinuated between the clavicle and inferior side of the tumour. The tumour presents an even surface, gives to pressure a slight degree of elasticity, but a small quantity of its contents only can be forced into the artery. It may be seen to pulsate from the opposite part of the room, and is attended with the tremulous motion and the hissing noise so characteristic of aneurism. These peculiarities are more strikingly evident immediately below the subclavian muscle and under the inferior fibres of the pectoralis major; the course of the subclavian artery, towards the axilla and beyond the aneurismal sac. The patient feels some pain in the tumour when he coughs; is indolent and sleepy; complains still of the tingling sensation in the fingers, and numbness in the middle and outer part of the fore-arm, and more especially when he walks and hangs his arm by his side. Naturally costive, in all other respects is perfectly well.

The operation for aneurism could not be, without great rashness, in this case attempted: first, because the tumour appeared to spring from the artery before it had emerged from between the scaleni muscles;* and, secondly, because a ligature cannot be depended upon so near the probable

* Indeed, we find Professor Colles, of Dublin, saying, "This operation, difficult on the right, must be deemed absolutely impracticable on the left subclavian artery: for the great depth from the surface at which this vessel is placed; the direct course which it runs in ascending to the top of the pleura; the sudden descent which it makes from this to sink under the protection of the clavicle, and the danger of including in the same ligature the eighth pair of nerves, the internal jugular vein, or the carotid artery, which all run close to, and nearly parallel with this artery; these all constitute such a combination of difficulties, as must deter the most enterprising surgeon from undertaking this operation on the left side."—Edinburgh Medical and Surgical Journal, p. 23.

burst burst* of this great blood-vessel, the coats of which might also have undergone a morbid change.

The following was the plan of treatment which I adopted in this case, and which, I am happy to say, was attended with success.

Before I proceed, I will observe, that although I saw my patient always once, and generally twice, a week, from the commencement to nearly the final termination of the case, yet, without being tedious, I trust that the underwritten particulars will be found sufficiently minute to enable the reader to draw his own conclusions thereon.

February 21st.—Bled to two pints. To take a grain of digitalis three times a-day; low diet.

29th.—No alteration. Bled to two pints. Continue the digitalis.

March 12th.—Pulse 80 and not so full. In other respects the same. Bled to two pints. Continue the digitalis; to take ten grains of Extr. Colocynth cum Hydr. Submur. gr.iij. occasionally.

28th.—Complains of soreness over the tumour; no other alteration. Bled to twenty-four ounces.

April 5th.—Pulse 75, and more compressible. The digitalis and bleedings have not, however, produced that sensible effect on the circulation and constitution which might have been expected. Bled to two pints. Repeat the digitalis.

May 13th.—Pulse 50, slightly irregular, and easily yielding to pressure. Patient reduced in flesh and much weakened. Omit the digitalis, but continue the Cathartic occasionally.

The circulation being now favourable to the obliteration of the opening into the aneurismal sac, by the judicious application of pressure, I embraced the first opportunity of making that trial.

Considering, however, that the *sudden* compression of an artery seldom fails to increase its action, I thought it right to proceed in this respect by stratagem. I, therefore, determined to begin my pressure by only affording a tolerable degree of support to the tumour, increasing it gradually as circumstances might indicate. For this purpose, I contrived a small oblong doe-skin bag, firmly stuffed with horse-hair, rising towards the centre like a graduated com-

---

* The reader will perceive, that I am disposed to attribute this case to great muscular exertion, in which the artery was ruptured, the cellular membrane forming the aneurismal sac according to Scarpa.

press, and having two broad bands and buckles. The central part of the compress being applied to the superior and anterior surface of the tumour, one band was passed obliquely across the breast, under the right axilla, and brought over the back, was buckled to the upper edge of the compress at the superior costa of the scapula; the other band was passed under the left axilla and met its fellow in the opposite direction. This compression received an additional support by a long calico roller.

30th.—Pulse 70; tumour rather sore; skin hot. In other respects as usual. Bled to 20 ounces. To take a grain of digitalis, with ten grains of nitrate of potass, three times a-day. Apply six leeches to the tumour.

June 25th.—Secretions increased; soreness removed; no obvious alteration in the tumour; pulse 55. Continue all the remedies.

30th.—Pulse 48, and intermitting; nausea, and great debility; perspires much. Omit the digitalis.

August 14th.—Pulse 75, and full; the tumour is rather larger; complains of strong beating on the right side of the neck and left breast—in the course of the subclavian artery beyond the tumour. Bled to 24 ounces; felt extremely languid and faintly immediately after this bleeding. Has repeated the application of the leeches twice a-week ever since the 30th of May; says they remove the fulness of the parts.

21st.—Felt a dull pain and numbness about the scapula and axilla, in addition to the same sensations in the arm of the affected side; pulse stronger; slight heat and thirst, with a furred tongue. Bled to 16 ounces. Repeat the digitalis and nitrate of potass. To take ʒifs of the sulphate of magnesia in two wine-glassfuls of water, every morning before breakfast.

29th.—Increased action of the vessels; heat and thirst, &c. complained of on the 14th and 21st inst. have gradually abated. Continue.

September 8th.—Pulse 47, and languid; greatly reduced in flesh and strength; perspires profusely; head-ach. Omit the digitalis, &c. The pressure has been gradually increased, but without producing any apparent good.

16th.—Pulse 60, and regular; no head-ach or profuse perspirations; the tumour appears larger.

22d.—Complains again of the same pain about the scapula. No further alteration. Repeat the leeches and cathartic pills.

25th.—Pulse 76, and full; pain again in the shoulder and breast of the affected side; acquires strength and flesh; bled to 24 ounces; repeat leeches, pills, and digitalis.

30th.—Pulse 57; tumour enlarges; the superincumbent skin somewhat turgid. I discovered the whizzing noise in the right carotid; says the leeches remove the soreness and fulness in and about the tumour. Continue.

October 15th.—Patient's strength and flesh much reduced; pulse 48, and weak, but regular; slight head-ach and nausea. Omit the digitalis, &c.

November 12th.—The tumour has been increasing since the 16th of September, is about the size and shape of a half-pint breakfast cup, has risen an inch above the anterior surface of the clavicle, and has raised that bone somewhat out of its natural situation. Pulse in the left wrist affected by the tumour, being weaker and more irregular than in the other arm. Temperature of both extremities the same. Repeat leeches and the saline purgative.

December 5th.—Tumour still larger. He feels more restless at night, and more pain and numbness in the shoulder and fore-arm. Repeat the digitalis, and continue the other remedies.

January 1st, 1814.—Tumour stationary as to size. From this time the case went on without any particular alteration, the remedies being occasionally employed as the circulation required them, till

April 15th,—When the tumour did not pulsate so strong; it had become tenser, and the pains in the shoulder, &c. had ceased. The superincumbent skin is moveable, and of its natural colour.

May 10th.—No alteration since the last report. Continue the digitalis, increase the pressure, and repeat the leeches. Pil. Colocynth. cum Cal.

June 1st.—Tumour less, and remarkably tense; pulsation scarcely perceptible; tremulous motion and hissing noise not discoverable; no pain or soreness in or about the tumour; has the perfect use of the arm, without numbness and the tingling sensation in the fingers, which he has, more or less, complained of from the commencement; feels no pressure against the clavicle, but, now and then, a tingling and sense of heat about the scapula; pulse 78, and regular. The digitalis seems to have lost its effect in a great measure, the patient taking it for a much longer time than usual without being so sensibly affected. Continue digitalis, leeches, and pressure. The bowels have been constantly kept in a regular state by the alternate use of the drastic and saline purgatives. Bled to 14 ounces.

8th.

8th.—No pulsation in the tumour, and rather less. Continue the remedies.

July 4th.—Pulse 67, and full; tumour lessens. Omit digitalis. Bled to 16 ounces.

August 10th.—Pulse regular; tumour diminishes slowly. Bled to 14 ounces.

September 5th.—General health tolerably good; has regained some strength and flesh; pulse natural; tumour gradually lessens. Continue the pressure, leeches, and Pil. Colocynth. cum Cal.

October 1st.—Bled to 12 ounces. Continue. From this period the tumour rapidly diminished, and, in the latter end of December, no trace of it was to be discovered.

*Concluding observations.*—The depleting and sedative plan of treatment was, in this case, persisted in for more than a year and half. The force, as well as the rapidity, of the circulation, was thus constantly kept below par, and at times exceedingly weak. Meanwhile, the pressure directed the blood into the collateral vessels. Hence, little or no enlargement of the aneurismal tumour for many months. At length the tumour gradually enlarged, then remained stationary as to size, and next acquired an incompressible tenseness. The artificial pressure was now more particularly increased; and the other remedies were occasionally repeated. The pulsatory motion ceased. The tumour at first gradually, then rapidly, lessened, and finally disappeared. The arm soon recovered its wonted power, and the body its former strength and plumpness.

But, whether the cure of this aneurism is in any *very great* degree to be attributed to artificial pressure, is to me extremely questionable:—1st, because *a small portion* only of the contents of the sac could be thrust back into the artery; 2dly, because the pressure was not for *a long time* attended with any important advantage; and 3dly, because the tumour did not cease beating, nor diminish in size, *till it had acquired a great degree of tension.* I believe that the large bleedings so often repeated, and the sedative plan so actively and perseveringly followed up, by preventing the usual flow of blood into the sac, enabled its contents to undergo a more perfect and general state of coagulation, and that this led to the increased tension of the tumour. And, also, that this state of tension, or natural compression, combined with artificial pressure, had the effect of damming up the opening into the aneurismal sac, by forcing and retaining the coagulated blood against the ruptured side of the artery, during which coagulable lymph was thrown out, and, becoming organised, effected the complete obliteration of the aperture.

The

The following case, illustrative of one of the consequences of venesection, is not without interest.

Mr. John Broadribb, æt. 28, a generous liver, complained of pain and uneasiness in the left breast, which was increased by a deep inspiration. He laboured under no other particular inconvenience. I, at this time, happened to call at his father's house on my way from Bristol to Frome, when my advice was solicited. I recommended him to lose some blood, which was immediately complied with. The bleeding, which was from the left arm, soon afterwards gave him relief. A few hours, however, had scarcely elapsed, when (as I subsequently learnt) great pain was felt in the fore-arm and shoulder. These symptoms, as they did not alarm the family, were not attended to for *four days!* during which, extensive inflammation and partial delirium took place. In this state he was conveyed in a chaise to a surgeon and relative in the adjoining village, whose illness confined him to his bed. This gentleman stated it as his opinion that the inflammation was the immediate though unavoidable consequence of the bleeding. A respectable surgeon in the neighbourhood was now sent for, who concurred in the same opinion, and afterwards informed me that an inflammation of the lymphatics had extended up the fore-arm, and over the shoulder and left breast, with considerable "erisipelatous appearances;" that this had proceeded to a most violent degree, attended with delirium and the concomitant symptoms; and that gangrene and death followed.

*Note.*—The orifice healed kindly and rapidly, and was not attended with the slightest inconvenience. No discoloration of the skin was apparent at the time of bleeding. It must not be omitted that some of his family are subject to erisipelatous attacks.

JOHN C. YEATMAN,

*Member of the Royal College of Surgeons, &c.*

